# Identification and Characterization of Wilt and Salt Stress-Responsive MicroRNAs in Chickpea through High-Throughput Sequencing

**DOI:** 10.1371/journal.pone.0108851

**Published:** 2014-10-08

**Authors:** Deshika Kohli, Gopal Joshi, Amit Atmaram Deokar, Ankur R. Bhardwaj, Manu Agarwal, Surekha Katiyar-Agarwal, Ramamurthy Srinivasan, Pradeep Kumar Jain

**Affiliations:** 1 NRC on Plant Biotechnology, IARI Campus (PUSA), New Delhi, India; 2 Department of Botany, North campus, University of Delhi, Delhi, India; 3 Department of Plant Molecular Biology, South Campus, University of Delhi, Delhi, India; National Key Laboratory of Crop Genetic Improvement, China

## Abstract

Chickpea (*Cicer arietinum*) is the second most widely grown legume worldwide and is the most important pulse crop in the Indian subcontinent. Chickpea productivity is adversely affected by a large number of biotic and abiotic stresses. MicroRNAs (miRNAs) have been implicated in the regulation of plant responses to several biotic and abiotic stresses. This study is the first attempt to identify chickpea miRNAs that are associated with biotic and abiotic stresses. The wilt infection that is caused by the fungus *Fusarium oxysporum* f.sp. *ciceris* is one of the major diseases severely affecting chickpea yields. Of late, increasing soil salinization has become a major problem in realizing these potential yields. Three chickpea libraries using fungal-infected, salt-treated and untreated seedlings were constructed and sequenced using next-generation sequencing technology. A total of 12,135,571 unique reads were obtained. In addition to 122 conserved miRNAs belonging to 25 different families, 59 novel miRNAs along with their star sequences were identified. Four legume-specific miRNAs, including miR5213, miR5232, miR2111 and miR2118, were found in all of the libraries. Poly(A)-based qRT-PCR (Quantitative real-time PCR) was used to validate eleven conserved and five novel miRNAs. miR530 was highly up regulated in response to fungal infection, which targets genes encoding zinc knuckle- and microtubule-associated proteins. Many miRNAs responded in a similar fashion under both biotic and abiotic stresses, indicating the existence of cross talk between the pathways that are involved in regulating these stresses. The potential target genes for the conserved and novel miRNAs were predicted based on sequence homologies. miR166 targets a HD-ZIPIII transcription factor and was validated by 5′ RLM-RACE. This study has identified several conserved and novel miRNAs in the chickpea that are associated with gene regulation following exposure to wilt and salt stress.

## Introduction

MicroRNAs (miRNAs) are small, endogenous, non-coding RNAs that are present in animals, plants and some viruses. These RNAs participate in the regulation of target genes by binding to complementary mRNAs, resulting in either their cleavage or translational repression. miRNAs are involved in diverse processes in different organisms, including developmental timing in worms, cell death and fat metabolism in flies, hematopoiesis in mammals and leaf development, floral patterning and environmental stress responses in plants [1].

*MIRNA* genes are transcribed as independent transcriptional units by RNA polymerase II enzymes to generate primary miRNAs (pri-miRNAs). pri-miRNAs form imperfect folded structures that are processed by Dicer-like1 nuclease (a member of the RNase III endonuclease family) to precursor miRNAs (pre-miRNAs). The secondary structures of these precursors are well conserved in plants. The pre-miRNA contains a miRNA-star miRNA (miRNA*) intermediate duplex from which the miRNA* eventually is degraded. However, recent studies have revealed the higher accumulation of miRNA* under certain conditions in plants, indicating the probable role of miRNAs in modulating plant growth and development [2]. Mature miRNAs are 19 to 24 nucleotides (nt) in length and interact with an RNA-induced silencing complex (RISC) to cleave specific target mRNAs or inhibit their translation. This complementarity plays an important role in determining the fate of the mRNA. When the complementarity between the miRNA and mRNA is perfect or near perfect, the mRNA is cleaved; however, if there are many mismatches between them, translational repression occurs. There are also instances in which miRNAs and mRNAs with perfect complementarities lead to the repression of translation and not to the usual cleavage.

The first identified miRNAs were lin4 and let7 in *Caenorhabditis elegans*, which is a model nematode [3], [4]. The first plant miRNAs were identified in Arabidopsis [5] and later in other plants. Currently, 7,321 mature miRNAs have been reported in 72 plant species (miRBase version 20) [6]. Among dicots, the maximum number of miRNAs occurs in the legume family (1,460), followed by Brassicaceae (863). Although the legume family has the best representation in terms of the number of miRNAs, chickpea is a notable omission from the list.

Chickpea (*Cicer arietinum*) is the world's second most widely grown legume and is cultivated in more than 40 countries. The Indian subcontinent is the principal chickpea-producing and consuming region, contributing almost 70% of the world's total production [7]. Chickpea seeds are a rich source of protein and starch for the human population and the records of chickpea cultivation date back to 6,000 BC. Globally, chickpea is grown on 11.5 million hectares (ha) to produce 10.4 million tons with an average yield of approximately 0.9 t/ha, which is far below its yield potential of 6 t/ha under optimal growth conditions [7]. The disparity between the actual and potential yields can be explained by large numbers of biotic and abiotic stresses that adversely affect its productivity. Among the biotic stresses, wilt infection that is caused by the fungus *Fusarium oxysporum* f.sp. *ciceris* is a major concern. Abiotic stress conditions, such as terminal drought and salt stress, also lead to major losses. ICC4958 is a drought tolerant chickpea cultivar and gets affected at terminal drought, which occurs at the pod filling and seed-developing stage of the crop [8], [9]. However, recent studies on salinity tolerance and ion accumulation in chickpea have revealed it as a highly sensitive crop to salinity when compared to other species in cropping systems [10], [11]. Thus, salinity is another major constraint in chickpea yield. A better understanding of genes and their interactions with the environment can play a very important and determinant role in tackling these stress conditions. The recently available transcriptome and genome sequences that have been reported by independent groups are important resources that will facilitate the attainment of these goals in the chickpea [12], [13], [14]. Hu et al. (2013) identified 28 potential miRNA candidates belonging to 20 families from 16 ESTs and 12 GSSs in the chickpea using a comparative genome-based computational analysis [15]. A total of 664 miRNA targets were predicted, including genes encoding transcription factors (TFs) in addition to those that function in the stress response, signal transduction, methylation and a variety of other metabolic processes. These findings lay the foundation for the elucidation of miRNA function in the development and stress responses of the chickpea.

miRNAs have been discovered primarily using direct cloning and bioinformatic approaches. All of the miRNAs in plants have been identified via the cloning of small RNAs or a computational approach, in which the homologs of known miRNAs are searched. We have generated small RNA libraries corresponding with the control conditions, Fusarium wilt infection and salt stress, which were sequenced using the Illumina sequencing platform to identify miRNAs in the chickpea. This study is the first report in which small RNA libraries have been constructed and sequenced to identify miRNAs in the chickpea.

## Results

### Sequence analyses

**Figure 1:**
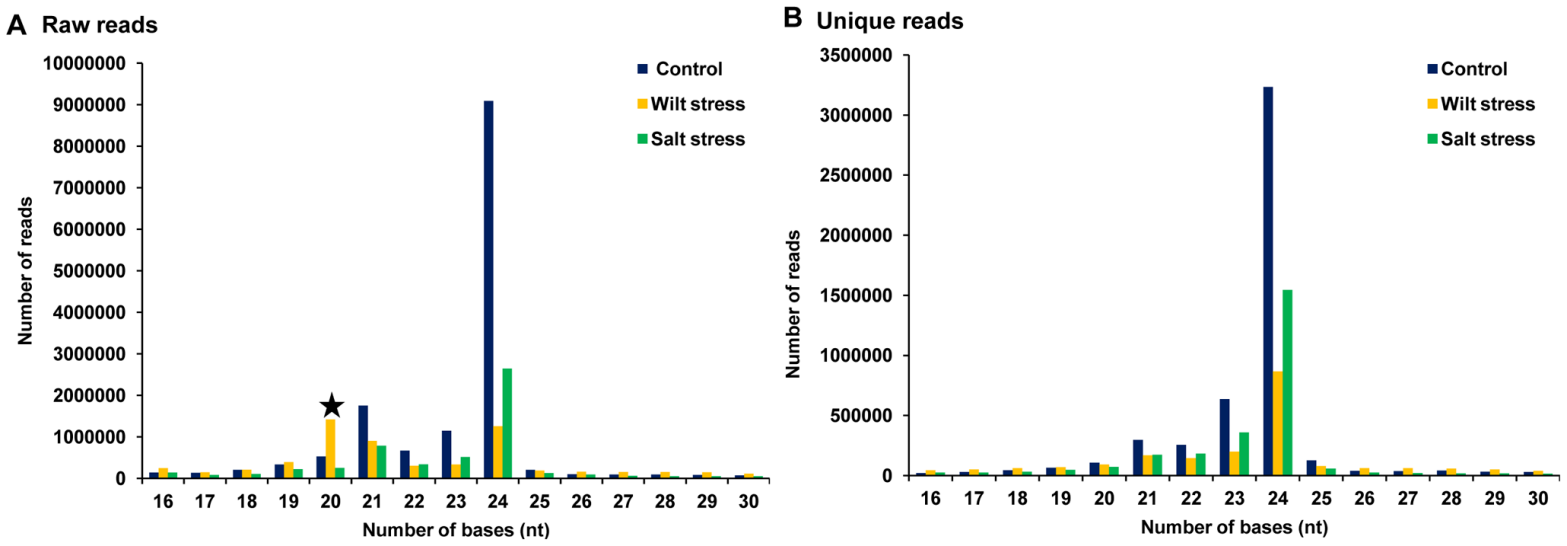
Length distribution of small RNA population. Size distributions of the miRNAs in the three chickpea libraries. In the wilt stress library, 20 nt miRNAs are more frequent than 24 nt miRNAs. However, in the other two libraries 24 nt miRNAs are more frequent.

**Figure 2:**
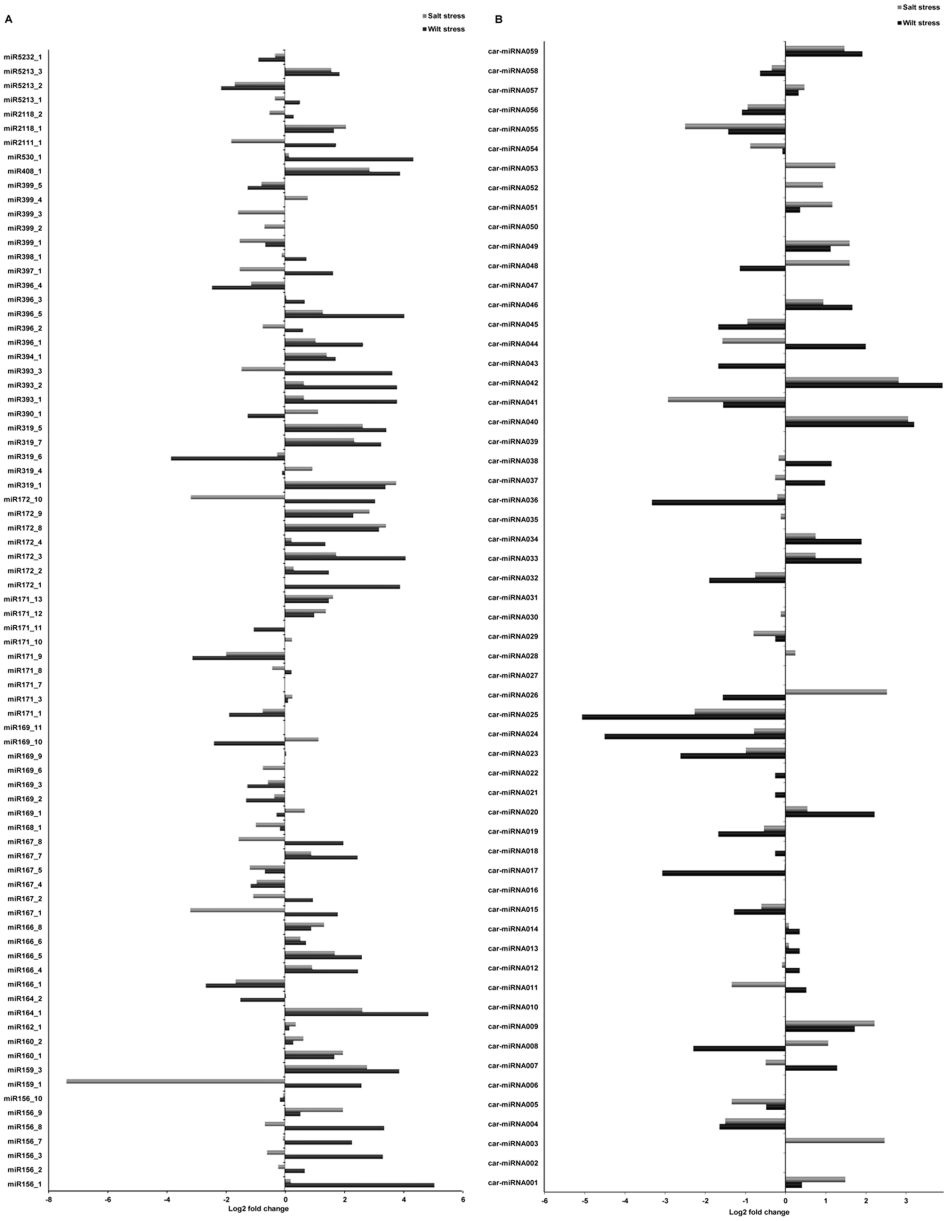
Differential expression patterns of chickpea miRNAs under wilt and salt stresses. (A) Conserved miRNAs, (B) novel miRNAs.

Three separate small RNA libraries that were constructed from the total RNA of the control, Fusarium wilt-infected and salt-stressed plants were subjected to Illumina Solexa sequencing. This sequencing generated 29,170,463 raw reads, which after processing by UEA sRNA workbench 2.4- Plant version sequence file pre-processing tool (http://srna-tools.cmp.uea.ac.uk/), produced approximately 12,135,571 total unique reads (Table 1). After removing the adaptor sequences, filtering the low-quality tags and eliminating the t/rRNA sequences, the putative small RNA population accounted for approximately 88.5%, 79.1% and 78.4% in the control, wilt-infected and salt-stressed libraries, respectively (Figure S1). The majority of small RNAs (approximately 50%) from the control and salt-stressed libraries were 24 nt in length (Figure 1), which is similar to other plant species, such as *Arabidopsis thaliana*, *Solanum lycopersicum* and *Medicago truncatula* [16], [17], [18]. Notably, in the wilt-infected library, small RNAs that were 20 nt in length accounted for 20% of the population, but when unique reads were analyzed, the small RNA distribution revealed that 24 nt was the major size class. Similar patterns have been reported in cucumber and soybean [19], [20]. In soybean, the unique and redundant sequence classes possessed 24 nt and 23 nt long small RNAs, respectively, as the most abundant sequences. For the differential expression analysis, the total numbers of miRNA reads in each given sample were normalized as transcripts per million, and the fold changes between the treated and control samples were calculated. Out of 122 conserved miRNAs, 44 were upregulated in response to wilt stress, but in the case of salt stress, the number of down regulated miRNAs was greater than that which was observed in response to wilt stress. However, the differential expression of novel miRNAs under both of these stresses showed relatively similar patterns, with approximately 60% of the miRNAs being down regulated under either wilt and/or salt stress (Figure 2a, b).

**Table 1:** Distribution of the sequenced reads in the control, wilt- and salt-stressed chickpea libraries.

Library	Control		Wilt Stress		Salt Stress	
	Total	Unique	Total	Unique	Total	Unique
Total number of sequences	15,744,289	6,103,870	7,007,282	2,677,947	6,418,892	3,353,754
Sequences remaining after 3′ adaptor removal (TCGTAT)	14,782,514	5,033,168	6,412,190	2,130,060	5,761,024	2,683,044
Sequences remaining after size-range filtering (16 to 30 nt)	14,689,499	5,005,568	6,192,469	2,054,560	5,584,159	2,627,840
SSR/TR	1414	757	222	216	359	307
t/rRNA	720,912	35,136	645,529	37,632	538,049	32,987
**Putative small RNA population**	**13,940,841**	**4,946,095**	**5,546,371**	**2,016,408**	**5,034,576**	**2,584,961**

### Identification of conserved miRNAs in chickpea

The unique reads that were obtained from the miRCat analysis tool (UEA small RNA workbench) were mapped to the miRNAs that were available in miRBase version 18 (http://www.mirbase.org/) [21], [22], [23]. The small RNA sequences that matched the known miRNAs from the miRBase database were identified as conserved miRNAs in the chickpea. The sequence analyses revealed the presence of 122 miRNAs belonging to 25 conserved families. The most abundant family was miR156 with 14 members. Among the others, miR171 (12 members), miR169 and miR172 (9 members each), miR166 and miR167 (8 members each), miR319 and miR399 (6 members each) and miR396 (5 members) were present. The remaining miRNA families had less than five members, with some families, such as miR530, miR162, miR5232 and miR408, being represented by only one member (Table 2; Table S1). In a recent report of the chickpea genome, the sequences of 20 unique miRNA families were reported, of which MIR169_2 and MIR159 were the most abundant [14]. In our study, out of 25 conserved miRNAs families, 16 possessed miRNA* sequences, thus providing additional evidence in support of the authenticity of the miRNAs. However, no miRNA* sequences were obtained for miR2111, miR162, miR164, miR390, miR394, miR397, miR530, miR408 or miR5213. Detailed information regarding the precursor structures of the conserved miRNAs is provided in Table S2.

**Table 2:** Conserved miRNAs in chickpea.

miR_ID	miR Family	Sequence	L	Conserved in					miRNA*	Start	End	PL	MFE	Adjusted	Hairpin
				gma	mtr	vun	ath	os	Y/N					MFE	G/C%
miR156_1	mir156	UUGACAGAAGAGAGAGAGCAC	21	+	-	-	-	-	N	52925	52945	85	-41.8	-49.176468	44.705883
miR156_2		UUGACAGAAGAUAGAGGGCAC	21	+	-	-	-	-	N	17629788	17629808	109	−42.6	−39.08257	38.532112
miR156_3		UGACAGAAGAGAGUGAGCAC	20	+	+	+	+	+	Y	44874629	44874648	92	−53.1	−57.71739	44.56522
miR156_4		UGACAGAAGAGAGUGAGCAC	20						Y	35818615	35818634	91	−52.8	−58.021973	43.956043
miR156_5		UGACAGAAGAGAGUGAGCAC	20						N	35226603	35226622	85	−39.6	−46.588234	42.352943
miR156_6		UGACAGAAGAGAGUGAGCAC	20						N	1284221	1284240	145	−65.1	−44.896553	37.931034
miR156_7		UGACAGAAGAGGGUGAGCAC	20	+	+	+	-	-	N	3228450	3228469	77	−30.7	−39.870132	32.467533
miR156_8		UGACAGACGAGAGUGAGCAC	20	+	-	+	-	-	N	7424	7443	91	−52.7	−57.912086	42.857143
miR156_9		UUGACAGAAGAUAGAAAGCAC	21	+	+	+	+	-	N	2202	2222	94	−42.7	−45.42553	39.361702
miR156_10		UUGACAGAAGAUAGAGAGCAC	21	+	+	+	-	-	Y	134	154	155	−60.3	−38.903225	32.258064
miR156_11		UUGACAGAAGAUAGAGAGCAC	21						Y	2528278	2528298	81	−47.1	−58.148148	39.506172
miR156_12		UUGACAGAAGAUAGAGAGCAC	21						N	25200808	25200828	82	−45.9	−55.97561	32.92683
miR156_13		UUGACAGAAGAUAGAGAGCAC	21						N	14260671	14260691	100	−46.4	−46.4	34
miR156_14		UUGACAGAAGAUAGAGAGCAC	21						N	14160024	14160044	119	−51.1	−42.941174	35.294117
miR159_1	mir159	UUUGGAUUGAAGGGAGCUCUA	21	+	+	-	+	-	Y	14884652	14884672	195	−97.2	−49.846153	38.46154
miR159_2		UUUGGAUUGAAGGGAGCUCUA	21						Y	11393460	11393480	195	−94.2	−48.30769	38.97436
miR159_3		AUUGGAGUGAAGGGAGCUCCA	21	+	+	-	-	-	N	13935752	13935772	188	−83.4	−44.361702	41.489365
miR160_1	mir160	UGCCUGGCUCCCUGAAUGCCA	21	-	+	-	-	+	N	6658138	6658158	85	−42.8	−50.352943	40
miR160_2		UGCCUGGCUCCCUGUAUGCCA	21	+	+	+	+	+	Y	32984804	32984824	86	−45.6	−53.023254	48.837208
miR160_3		UGCCUGGCUCCCUGUAUGCCA	21	+	+	+	+	+	Y	10315830	10315850	86	−47.1	−54.76744	47.674416
miR160_4		UGCCUGGCUCCCUGUAUGCCA	21	+	+	+	+	+	N	19971530	19971550	86	−48.8	−56.744186	48.837208
miR162_1	mir162	UCGAUAAACCUCUGCAUCCAG	21	+	+	+	+	+	N	7679640	7679660	124	−47.5	−38.30645	42.741936
miR164_1	mir164	UGGAGAAGCAGGGCACAUGCU	21	-	+	-	-	-	N	33442678	33442698	75	−34.34	−45.786667	45.333336
miR164_2		UGGAGAAGCAGGGCACGUGCA	21	+	+	+	+	+	N	20759462	20759482	175	−68.1	−38.914284	39.42857
miR164_3		UGGAGAAGCAGGGCACGUGCA	21						N	38007519	38007539	86	−37.3	−43.372093	41.860466
miR164_4		UGGAGAAGCAGGGCACGUGCA	21						N	7727761	7727781	118	−45.93	−38.92373	47.457626
miR166_1	mir166	UCGGACCAGGCUUCAUUCCCC	21	+	+	-	+	+	Y	47802209	47802229	101	−42.15	−41.732674	47.524754
miR166_2		UCGGACCAGGCUUCAUUCCCC	21						Y	34613199	34613219	80	−41.9	−52.375	47.5
miR166_3		UCGGACCAGGCUUCAUUCCCC	21						N	2982935	2982955	98	−46.2	−47.142857	41.836735
miR166_4		UCGGACCAGGCUUCAUUCCCG	21	+	-	-	-	-	Y	609780	609800	163	−59.7	−36.625767	34.969322
miR166_5		UCGGACCAGGCUUCAUUCCCU	21	-	-	-	-	+	N	10985854	10985874	168	−60.8	−36.190475	39.88095
miR166_6		UCGGACCAGGCUUCAUUCCUC	21	-	+	-	-	+	N	45773545	45773565	94	−39.78	−42.31915	46.80851
miR166_7		UCGGACCAGGCUUCAUUCCUC	21						N	788963	788983	99	−48.07	−48.555557	40.40404
miR166_8		UCUCGGACCAGGCUUCAUUCC	21	+	-	-	-	-	Y	609782	609802	163	−59.7	−36.625767	34.969322
miR167_1	mir167	UGAAGCUGCCAGCAUGAUCU	20	+	+	-	-	-	N	32102772	32102791	71	−37.4	−52.676056	39.43662
miR167_2		UGAAGCUGCCAGCAUGAUCUA	21	+	+	-	+	+	N	30873622	30873642	161	−67.2	−41.73913	37.8882
miR167_3		UGAAGCUGCCAGCAUGAUCUA	21						N	1691349	1691369	101	−48.9	−48.415844	39.603962
miR167_4		UGAAGCUGCCAGCAUGAUCUG	21	+	+	-	-	+	N	34009600	34009620	210	−79.6	−37.90476	32.380955
miR167_5		UGAAGCUGCCAGCAUGAUCUGA	22	+	-	-	-	-	Y	5038949	5038970	102	−49.1	−48.137253	39.215687
miR167_6		UGAAGCUGCCAGCAUGAUCUGA	22						Y	38728619	38728640	108	−44.93	−41.601852	48.148148
miR167_7		UGAAGCUGCCAGCAUGAUCUGG	22	-	-	-	+	-	Y	2509	2530	158	−57.8	−36.58228	42.405064
miR167_8		UGAAGCUGCCAGCAUGAUCUUA	22						N	57870929	57870950	75	−36.3	−48.4	45.333336
miR168_1	mir168	UCGCUUGGUGCAGGUCGGGAA	21	+	+	+	+	-	Y	7328597	7328617	136	−65.9	−48.455883	55.88235
miR169_1	mir169	AGCCAAGGAUGACUUGCCGG	20	+	+	+	+	+	N	19659099	19659118	86	−38.3	−44.53488	46.51163
miR169_2		CAGCCAAGGAUGACUUGCCGA	21	+	+	-	+	+	N	10422873	10422893	200	−83.7	−41.85	34
miR169_3		CAGCCAAGGAUGACUUGCCGG	21	+	+	+	+	+	N	2353405	2353425	95	−40.7	−42.842106	46.31579
miR169_4		CAGCCAAGGAUGACUUGCCGG	21						N	3920369	3920389	135	−63.5	−47.037037	44.444447
miR169_5		CAGCCAAGGAUGACUUGCCGG	21						Y	2526196	2526216	119	−50.17	−42.15966	30.252102
miR169_6		CAGCCAAGGGUGAUUUGCCGG	21	+	+	-	-	-	N	19606166	19606186	134	−57.6	−42.985073	40.298508
miR169_9		GAGCCAAGGAUGACUUGCCGG	21	-	+	-	-	-	N	19659098	19659118	86	−38.3	−44.53488	46.51163
miR169_10		UGAGCCAGGAUGACUUGCCGG	21	-	+	-	-	-	Y	19609060	19609080	76	−37.4	−49.21053	42.105263
miR169_11		CAGCCAAGGAUAACUUGCCGG	21	+	+	+	+	+	N	4770	4790	94	−38.5	−40.957447	43.617023
miR171_1	mir171	UGAUUGAGCCGCGUCAAUAUC	21	-	+	-	-	-	N	14153792	14153812	102	−47.7	−46.76471	40.19608
miR171_3		UGAUUGAGCCGUGCCAAUAUC	21	+	+	-	+	+	Y	1004434	1004454	97	−49.6	−51.13402	36.082474
miR171_4		UGAUUGAGCCGUGCCAAUAUC	21						N	5540425	5540445	78	−34.4	−44.102566	34.615387
miR171_5		UGAUUGAGCCGUGCCAAUAUC	21						N	15207158	15207178	94	−40.2	−42.765957	39.361702
miR171_6		UGAUUGAGCCGUGCCAAUAUC	21						N	6250414	6250434	117	−45.7	−39.05983	30.769232
miR171_7		UGAUUGAGUCGUGCCAAUAUC	21	-	+	-	-	-	N	73	93	77	−32.6	−42.337658	35.064934
miR171_8		AGAUAUUGGUGCGGUUCAAUC	21	+	-	-	-	-	Y	36014922	36014942	102	−52.4	−51.37255	38.235294
miR171_9		CGAUGUUGGUGAGGUUCAAUC	21	+	-	-	-	-	Y	27995981	27996001	95	−39.8	−41.894737	40
miR171_10		UUGAGCCGCGCCAAUAUCAC	20	-	-	-	-	-	N	1567139	1567158	92	−41.5	−45.108696	47.826088
miR171_11		UUGAGCCGCGCCAAUAUCACU	21	+	-	-	-	-	Y	6636919	6636939	94	−42.6	−45.319145	39.361702
miR171_12		UUGAGCCGUGCCAAUAUCAC	20	-	-	-	-	-	N	14104770	14104789	85	−35.7	−42	36.47059
miR171_13		UUGAGCCGUGCCAAUAUCACA	21	+	-	-	-	-	N	5540422	5540442	78	−34.4	−44.102566	34.615387
miR172_1	mir172	AGAAUCCUGAUGAUGCUGCAG	21	-	+	-	-	-	N	33800439	33800459	134	−69.8	−52.089554	38.059704
miR172_2		AGAAUCUUGAUGAUGCUGCA	20	-	-	-	+	-	N	1018482	1018501	106	−48.7	−45.943398	40.56604
miR172_3		AGAAUCUUGAUGAUGCUGCAG	21	-	-	-	+	-	Y	1018481	1018501	106	−48.7	−45.943398	40.56604
miR172_4		AGAAUCUUGAUGAUGCUGCAU	21	+	+	+	+	+	Y	11960794	11960814	108	−47.4	−43.88889	29.62963
miR172_5		AGAAUCUUGAUGAUGCUGCAU	21						Y	28871865	28871885	112	−47.51	−42.419643	39.285713
miR172_6		AGAAUCUUGAUGAUGCUGCAU	21						N	11265321	11265341	82	−39.1	−47.682926	34.146343
miR172_8		GCAGCAGCAUCAAGAUUCACA	21	+	-	-	-	-	Y	2893096	2893116	184	−71.7	−38.96739	39.673912
miR172_9		GGAGCAUCAUCAAGAUUCACA	21	-	-	-	-	-	Y	2969008	2969028	126	−58	−46.031746	43.650795
miR172_10		GAAUCUUGAUGAUGCUGCAG	20	+	+	+	+	-	Y	2969100	2969119	124	−57.1	−46.048386	44.35484
miR319_1	mir319	UUGGACUGAAGGGAGCUCCCU	21	+	+	+	+	+	Y	4935256	4935276	216	−74.03	−34.273148	39.814816
miR319_2		UUGGACUGAAGGGAGCUCCCU	21						N	27801302	27801322	216	−93.6	−43.333332	35.185184
miR319_4		UUGGACUGAAGGGGCCUCUU	20	+	-	-	-	-	N	15212722	15212741	207	−90.71	−43.821255	44.927536
miR319_5		UGGACUGAAGGGGAGCUCCUUC	22	+	-	-	-	-	N	46353126	46353147	213	−90.2	−42.347416	35.21127
miR319_6		GAGCUUCCUUCAGUCCACUC	20	+	+	+	+	-	Y	31787184	31787203	199	−86.6	−43.517586	38.693466
miR319_7		UGGACUGAAGGGAGCUCCUUC	21	+	+	+	+	+	N	9436191	9436211	93	−49.3	−53.01075	47.31183
miR390_1	mir390	AAGCUCAGGAGGGAUAGCGCC	21	+	+	-	+	-	N	26531589	26531609	82	−47	−57.317074	43.90244
miR390_2		AAGCUCAGGAGGGAUAGCGCC	21						N	26325697	26325717	71	−39.4	−55.49296	40.84507
miR393_1	mir393	UCCAAAGGGAUCGCAUUGAUCC	22	+	-	-	+	-	N	30299002	30299023	121	−53.2	−43.96694	34.710743
miR393_2		UCCAAAGGGAUCGCAUUGAUCC	22						N	1754148	1754169	77	−32.4	−42.077923	28.57143
miR393_3		AUCAUGCUAUCCCUUUGGAUU	21	+	+	-	+	+	Y	34480633	34480653	141	−57.7	−40.921986	39.00709
miR394_1	mir394	UUGGCAUUCUGUCCACCUCC	20	+	-	-	+	+	N	49015076	49015095	129	−62.3	−48.294575	41.08527
miR394_2		UUGGCAUUCUGUCCACCUCC	20						N	31533893	31533912	67	−26.03	−38.85075	47.761192
miR394_3		UUGGCAUUCUGUCCACCUCC	20						N	8950170	8950189	125	−36.35	−29.079998	40.8
miR396_1	mir396	UUCCACAGCUUUCUUGAACUG	21	+	+	-	+	+	N	35366632	35366652	114	−45.6	v39.999996	39.473686
miR396_2		UUCCACAGCUUUCUUGAACUU	21	+	+	-	+	+	N	556766	556786	85	-35.9	-42.2353	35.294117
miR396_3		CUCAAGAAAGCUGUGGGAGA	20	+	+	-	-	-	Y	6590654	6590673	108	-47.9	-44.351852	37.962963
miR396_4		GCUCAAGAAAGCUGUGGGAGA	21	+	+	-	-	-	Y	6590654	6590674	108	-47.9	-44.351852	37.962963
miR396_5		UUCCACAGUUUUCUUGAACUG	21	+	+	-	+	+	N	31162290	31162310	121	-47.8	-39.50413	41.322315
miR397_1	mir397	UCAUUGAGUGCAGCGUUGAUG	21	+	-	-	+	+	N	1821127	1821147	153	-60.1	-39.281044	30.065361
miR398_1	mir398	UGUGUUCUCAGGUCGCCCCUG	21	+	+	-	-	+	N	33289822	33289842	80	−29.04	−36.3	50
miR398_2		UGUGUUCUCAGGUCGCCCCUG	21						N	33373257	33373277	108	−47.8	−44.25926	50
miR399_1	mir399	UGCCAAAGAAGAUUUGCCCCG	21	-	+	-	-	-	N	103	123	79	−33.4	−42.278484	41.772152
miR399_2		UGCCAAAGGAGAGCUGCCCUA	21	-	+	-	-	-	N	254628	254648	124	−48.8	−39.354836	35.48387
miR399_3		UGCCAAAGGAGAGCUGCCCUG	21	-	+	-	-	+	N	216204	216224	110	−47.8	−43.454544	36.363636
miR399_4		UGCCAAAGGAGAGCUGCUCUU	21	+	+	+	+	+	N	29007702	29007722	172	−60.23	−35.01744	29.651161
miR399_5		UGCCAAAGGAGAGUUGCCCUG	21	+	+	+	+	+	Y	441789	441809	103	−44.9	−43.592236	43.68932
miR399_6		UGCCAAAGGAGAGUUGCCCUG	21						N	12886367	12886387	89	−43.3	−48.651684	41.573032
miR408_1	mir408	AUGCACUGCCUCUUCCCUGGC	21	+	+	+	+	+	N	21952158	21952178	82	−36.6	−44.634144	51.219513
miR530_1	mir530	UGCAUUUGCACCUGCACUUUA	21	+	+	-	-	+	N	40319328	40319348	181	−79.3	−43.812157	38.674034
miR2111_1	mir2111	UAAUCUGCAUCCUGAGGUUUA	21	+	+	-	+	-	N	37594	37614	81	−30.5	−37.654324	33.333336
miR2111_2		UAAUCUGCAUCCUGAGGUUUA	21						N	93808	93828	66	−27.5	−41.666664	36.363636
miR2111_3		UAAUCUGCAUCCUGAGGUUUA	21						N	19125	19145	67	−33.1	−49.40298	31.343285
miR2111_4		UAAUCUGCAUCCUGAGGUUUA	21						N	37881	37901	81	−35.7	−44.074078	33.333336
miR2118_1	mir2118	UUACCGAUUCCACCCAUUCCUA	22	-	+	-	-	-	N	112	133	155	−41.2	−26.580647	40
miR2118_2		GGAUAUGGGAGGGUCGGUAAAG	22	+	-	-	-	-	Y	15537249	15537270	150	−60.54	−40.36	38
miR5213_1	mir5213	UACGUGUGUCUUCACCUCUGAA	22	+	-	-	-	-	N	16737686	16737707	119	−42.1	−35.37815	36.974792
miR5213_2		UACGUGUGUCUUCACCUCUGA	21	-	+	-	-	-	N	16737686	16737706	119	−42.1	−35.37815	36.974792
miR5213_3		UACGGGUGUCUUCACCUCUGA	21	-	+	-	-	-	Y	31172531	31172551	114	−47.7	−41.842106	38.59649
miR5232_1	mir5232	UACAUGUCGCUCUCACCUGGA	21	-	+	-	-	-	Y	29987519	29987539	167	−69.8	−41.79641	44.31138

gma- *Glycin max*; mtr- *Medicago tranculata*; vun- *Vigna unguiculata*; ath- *Arabidopsis thaliana*; os- *Oryza sativa*.

MFE- Minimum Folding Energy; L: Length; PL: Precursor Length.

### Identification of legume-specific miRNAs in chickpea library

**Figure 3:**
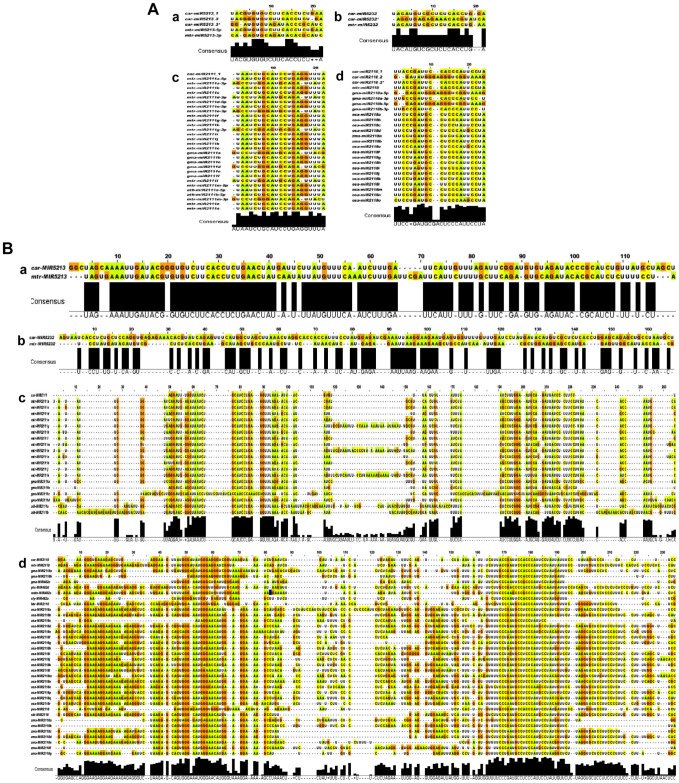
Multiple sequence alignments of legume-specific miRNAs. (A) Mature miRNAs, (B) precursor miRNAs. Four legume-specific miRNAs, including a) MIR5213, b) MIR5232, c) MIR2111and d) MIR2118, were used for the multiple sequence alignments by ClustalW2 in the different plants. car- *Cicer arietinum*, mtr- *Medicago truncatula*, gma- *Glycine max*, ath- *Arabidopsis thaliana*, osa- *Oryza sativa*, zma- *Zea mays*, sbi- *Sorghum bicolor*, sly- *Solanum lycopersicum*, hbr- *Hevea brasiliensis*, pvu*- Phaseolus vulgaris*, vun- *Vigna unguiculata*, ptc- *Populus trichocarpa* and mdm- *Malus domestica.*

We obtained four legume-specific miRNAs, including miR2111, miR2118, miR5213 and miR5232, in our libraries that were previously reported in another legume, Medicago [24], [25]. To date, miR5232 has only been reported in Medicago in a study involving miRNA regulation during arbuscular mycorrhizal symbiosis [25]. Accordingly, miR5232 may be a legume-specific miRNA that is involved in the biotic stress response. The multiple sequence alignment of the mature miRNAs in addition to the precursor sequences of these four legume-specific miRNAs revealed that they were most closely similar to Medicago and consequently has been conserved throughout evolution (Figure 3a, 3b). However, in recent studies, sequences that are similar to miR2118 have been reported in other non-leguminous plant systems, such as tomato and rice [26]. Apart from Fabaceae, miR2118 family members are most abundant in the Rutaceae and Solanaceae plant families [27]. Even the nomenclature of the miR2118 family is inconsistent in miRBase: the miR2118-like sequences have been disparately named miR482 (sly-miR482), miR5300 (as in tomato) and miR2809. The variation in the miR2118 sequence is species specific. Thus, miR2118 sequences in the chickpea are more similar with mtr-miR2118a [24] in comparison with other plant systems.

### Identification of novel miRNAs in chickpea

**Figure 4:**
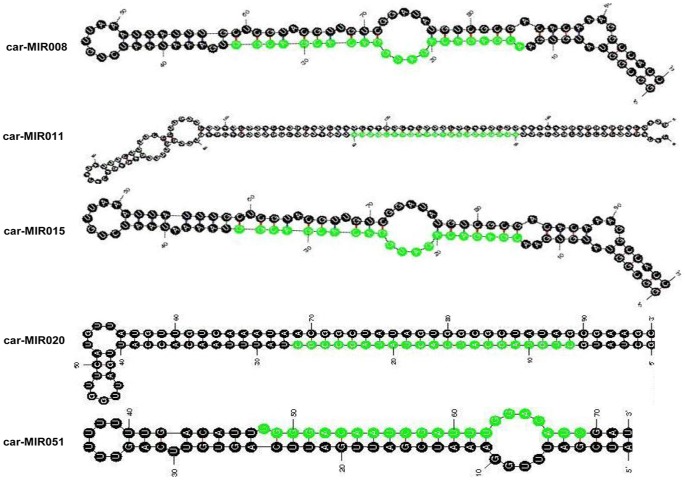
Predicted secondary structures of five validated novel miRNA precursors in chickpea using Mfold.

We identified 59 novel miRNAs using the miRCat module of the UEA sRNA workbench, which aligned the pooled reads from all three of the libraries to the chickpea genome (NCBI Genome: PRJNA175619) [13], the ESTs database from NCBI and transcriptome data from the chickpea transcriptome database [28], and applied prediction criteria for plant miRNAs [29] (Table 3; Table S3). The low abundance of novel miRNAs in our data supports the earlier notion of the lower expression levels of novel miRNAs compared with those of conserved miRNAs [30]. The precursor miRNA candidates were then tested using RandFold with a cutoff of 0.1. The minimum free energy that was required to form the predicted hairpin structure for the precursor was in the range of −97.2 to −26.03 Kcal/mol, which is similar to the values that were reported for the precursors of other plant species (Table S4). The secondary structures of the precursors of five validated novel miRNAs were evaluated using the Mfold software (Figure 4) [31]. The data analysis revealed the presence of miRNA* sequences for all of the 59 novel miRNAs of the chickpea. The miRNA* supports the release of the miRNA duplex from the predicted hairpin structure [32]; therefore, the presence of miRNA* sequences further supports the identity of these small RNA sequences in our libraries as novel miRNAs.

**Table 3:** List of novel chickpea miRNAs with their miRNA*.

New miRID	Sequence	L	miRNA*	Start	End	PL	MFE	Adjusted	Hairpin
								MFE	G/C%
car-miRNA001	AACCAGGCUCUGAUACCAUGA	21	AUGGUAUCAGGUCCUGCUUCA	20306919	20306939	87	−37.34	−42.91954	42.528736
car-miRNA002	AAGAUUGAUCUUGACCUUCUGC	22	UUAUGGCAUAAACAAGGAUAAU	588	609	93	−29	−31.182796	37.634407
car-miRNA003	AAGCAGGCUCUGAUACCAUGA	21	UGGUAUCAGGUCCUGCUUCA	696	716	95	−42.3	−44.526314	47.368423
car-miRNA004	AAUAGAUUGUCCAAUCGAUUGU	22	CAAUCGAUUUCCCAAUCGAUUU	343	364	159	−47.8	−30.062893	33.962265
car-miRNA005	AAUCACGGUGAGCCACUGUGA	21	AAUCACGGUGGCUCACCGUGA	251	271	91	−54.2	−59.56044	39.56044
car-miRNA006	ACCGGAAGCUGGGUUACGGUC	21	CGCGACCUAUACCCGGCCGU	598	618	199	−75.01	−37.693466	56.281406
car-miRNA007	ACGACUGUUACAUCAUACAAC	21	UGUAUGGUGCAACAGUCGCAG	23710719	23710739	137	−65.8	−48.029198	43.065693
car-miRNA008	ACGAGACAGAUGGACACGACGG	22	CGUACGUUGUCGGAUAUGUCGC	338	359	97	−28.5	−29.381443	47.42268
car-miRNA009	AGCGAUCUCGUACUAAACGAA	21	CUUCGAUAGGCGAGAGGUGUA	23987481	23987501	78	−24.9	−31.923077	47.435898
car-miRNA010	AGGAGAAAGUCUUUGCAACCG	21	UGUGUUGCUGAGACAUGCGCC	273099	273119	65	−21.7	−33.384617	52.307693
car-miRNA011	AUGGUUGAGAGGGUGACUUGA	21	AAGUCACUUUCUCAAUCUUA	1161	1181	155	−72.3	−46.645164	39.35484
car-miRNA012	CAGGGAACAGGCUGAGCAUGG	21	AUGCACUGCCUCUUCCCUGGC	171	191	87	−47.7	−54.827587	51.724136
car-miRNA013	CAGGGAACAGGCUGAGCAUGG	21	AUGCACUGCCUCUUCCCUGGC	149	169	87	−47.7	−54.827587	51.724136
car-miRNA014	CAGGGAACAGGCUGAGCAUGG	21	AUGCACUGCCUCUUCCCUGGC	285	305	87	−47.7	−54.827587	51.724136
car-miRNA015	CGAGACAGAUGGACACGACGG	21	CGUACGUUGUCGGAUAUGUCG	336	356	97	−28.5	−29.381443	46.391754
car-miRNA016	CGAUUGCGGCGACGUGGGCG	20	CUGCCCGCGACGUUGUGAGA	50	69	66	−32.8	−49.696968	57.575756
car-miRNA017	CGGAAUACAAGCUCUGUACCGGAA	24	CGGAACACUCUUCUGUACCGGAAA	18012181	18012204	116	−42.41	−36.560345	39.655174
car-miRNA018	CUGACUUAGCUUGUAGUCGAC	21	UAGUCGACUACAGAUGGGUGU	533	553	67	−30.5	−45.52239	46.268658
car-miRNA019	CUGGGUUGGGUCGAUCGGUCC	21	CACCGGUUGGCUCGUCCCUU	842	862	75	−32.8	−43.73333	65.33333
car-miRNA020	CUGUAGCAUCACUAUAGCCGC	21	CGGCUAUAGUGGCGCUAUAGC	37423709	37423729	95	−45	−47.368423	42.105263
car-miRNA021	GAAACGGGUAGCUGAGGGUU	20	CACUCUAAACAGCAGCUCCGU	580	599	113	−34.3	−30.353981	40.707962
car-miRNA022	GAAACGGGUAGCUGAGGGUU	20	CACUCUAAACAGCAGCUCCGU	499	518	113	−32.3	−28.584068	39.823006
car-miRNA023	GAAAUGGACGGCAAUGAAUCUA	22	UUGAAGGUUUUGCUGACCUUU	157	178	88	−26.52	−30.136364	42.045452
car-miRNA024	GAACGAGACAGAUGGACACGA	21	UACGUUGUCGGAUAUGUCGCGA	337	357	98	−29.9	−30.510204	45.918365
car-miRNA025	GAGUUCACUGUUGGAGAUGUGCCA	24	GCACAACUCCAACGGUGAACCCAC	30794763	30794786	220	−138.6	−63.000004	45.909092
car-miRNA026	GCCGGCCUGUCAGACCUAAUAGGC	24	UCAAGCCAUAGGCCUCUGACGGAC	9008149	9008172	162	−62.4	−38.518517	41.975307
car-miRNA027	GCGAAGCUAUCGUGCGUUGGAU	22	UUCGCACAAUUGGUCAUCGCG	138313	138334	95	−32.3	−34	50.526314
car-miRNA028	GGGUUGGGUCGAUCGGUCCA	20	CACCGGUUGGCUCGUCCCUU	677	696	69	−33.8	−48.985504	63.76812
car-miRNA029	GGGUUGGGUCGAUCGGUCCGCC	22	GGUGUGCACCGGUUGGCUCGU	18682982	18683003	69	−35.5	−51.449276	66.66667
car-miRNA030	GGGUUGGGUCGAUCUGUCCGCC	22	GGUGUGCACCGGUUGGCUCGU	619	640	69	−29.3	−42.463768	65.21739
car-miRNA031	GGUUGGGUCGAUCGGUUCGCCU	22	GUGUGCACCGGUCUGCUCGUC	383	404	69	−30.9	−44.782608	65.21739
car-miRNA032	GUUCUAGAUCGACGGUGGCAU	21	GUCACCACCGUCGUCUCGCA	103	123	66	−28.6	−43.333332	56.060608
car-miRNA033	GUUCUAGAUCGACGGUGGCAU	21	GUCACCACCGUCGUCUCGCA	111	131	66	−28.6	−43.333332	56.060608
car-miRNA034	GUUCUAGAUCGACGGUGGCAU	21	CACCACCGUCGUCUCGCAGCU	313	333	67	−27.6	−41.19403	56.71642
car-miRNA035	UAACUCUGAUGAAGUUGUGCA	21	GCUCAAUUUGUAUCUGGGACAU	147	167	61	−19.3	−31.639343	37.704918
car-miRNA036	UAAGUCGGUGACGUCUACGUAUAC	24	UCUAUACGGAAGAUGCAUGGACUA	21957421	21957444	82	−24.2	−29.512197	41.463413
car-miRNA037	UAGCGACACGGAACGUCCAAC	21	GGGGUUGGACGCUCCGUGCCA	215357	215377	112	−81.4	−72.67857	51.785713
car-miRNA038	UAUGUGAACGAGACAGAUGGA	21	UCGGAUAUGUCGCGACACAAA	342	362	98	−29.9	−30.510204	45.918365
car-miRNA039	UCAUAUUUGUUGGACAUUUGA	21	UCAAUGUAUUGAUGGGUAUGU	942	962	113	−29.7	−26.283186	38.938053
car-miRNA040	UCGCGUGAGUGAAGAAGGGCA	21	AGCUCUUUCGUCGAGUGCGCG	69427	69447	66	−23.3	−35.30303	50
car-miRNA041	UCGCUGUUGCGUUGGCGAUUA	21	AUCGCCACCGCAACAGCGAAG	21404687	21404707	114	−59.83	−52.482456	53.50877
car-miRNA042	UCGGACCAGGCUUCAUUCUUC	21	AAUGAGGUUUGAUCCAAGAUC	1317	1337	197	−57.8	−29.3401	38.071068
car-miRNA043	UGAACUAUUCGAUCUUCGUUC	21	GAUGAAGAUCAAACGGUUCAU	101	121	147	−67.8	−46.122448	32.65306
car-miRNA044	UGAAGCUGCCAGCAUGAUCUUA	22	AGAUCAUGUGGCAGUUUCACC	1047	1068	73	−36.4	−49.863018	47.945206
car-miRNA045	UGAUUGUAUAAUCGAUUAGGCA	22	AUUGGCAAAUCGAUUGUGCACA	26	47	121	−47.8	−39.50413	38.842976
car-miRNA046	UGAUUGUAUAAUCGAUUAGGCA	22	AUUGGCAAAUCGAUUGUGCACA	26	47	121	−47.8	−39.50413	38.842976
car-miRNA047	UGCCAAGCGCUGUAGUAGGUCA	22	AUAAGGCUUUUACAGCGCUUG	606	627	94	−50.1	−53.29787	44.68085
car-miRNA048	UGGACUAAAAUUCUGUUUGGAGAC	24	GUCCCCCAAGCAGAAUUUUGGUCC	30591970	30591993	218	−91.99	−42.197247	31.192661
car-miRNA049	UGGACUAAAAUUCUGUUUGGAGAC	24	GUCCCCCAAGCAGAAUUUUGGUCC	47072	47095	220	−95.82	−43.554543	31.818182
car-miRNA050	UGGAUUGAGAUCGAAUGGUGC	21	GACCGUCCGGUCUUGAUCCAAG	347	367	137	−60.2	−43.941605	37.956203
car-miRNA051	UGGGACAAUCGAUUUGGACAUC	22	UUGGAAAUCGAUUGAUUCAGUG	260	281	72	−22.4	−31.111109	34.72222
car-miRNA052	UGGUCUGUGAGAGACUGCACGGUA	24	UCGUGCUGGUCUGUGAGAGACUGC	52521	52544	96	−40.7	−42.395832	53.125
car-miRNA053	UGGUUGGGUCGAUCGGUCCGCC	22	GGUGUGCACCGGUUGGCUCGU	677	698	85	−37.1	−43.647057	63.529415
car-miRNA054	UGUGGAUGAUGCAGGAGCUGA	21	AGCUGCUGACUCAUUCAUUCA	27572322	27572342	152	−59.5	−39.144737	35.526314
car-miRNA055	UGUUGCAAUCGACCAGGACUAC	22	AGUCUUGAUCGAUGUAACUGA	385	406	79	−39.5	−50	32.911392
car-miRNA056	UUAACCAGGCUCUGAUACCAU	21	UGGUAUCAGGUCCUGCUUCA	21161303	21161323	88	−37.4	−42.5	50
car-miRNA057	UUCGAAUCCUGCCGUCCACGCC	22	AGUGGACGUGCCGGAGUGGUUA	29887519	29887540	94	−37.2	−39.574467	55.31915
car-miRNA058	UUGAGCCGCGUCAAUAUCUUG	21	CAAGGUAUUGGCGCGCCUCAA	24546067	24546087	92	−53.4	−58.04348	41.304348
car-miRNA059	UUGAUCUUUCGAUGUCGGCU	20	GUUUAGACCGUCGUGAGACA	988	1007	116	−45.4	−39.13793	50.86207

MFE: Minimum Folding Energy; L: Length; PL: Precursor Length.

### Expression patterns of known and novel miRNAs in chickpea

**Figure 5:**
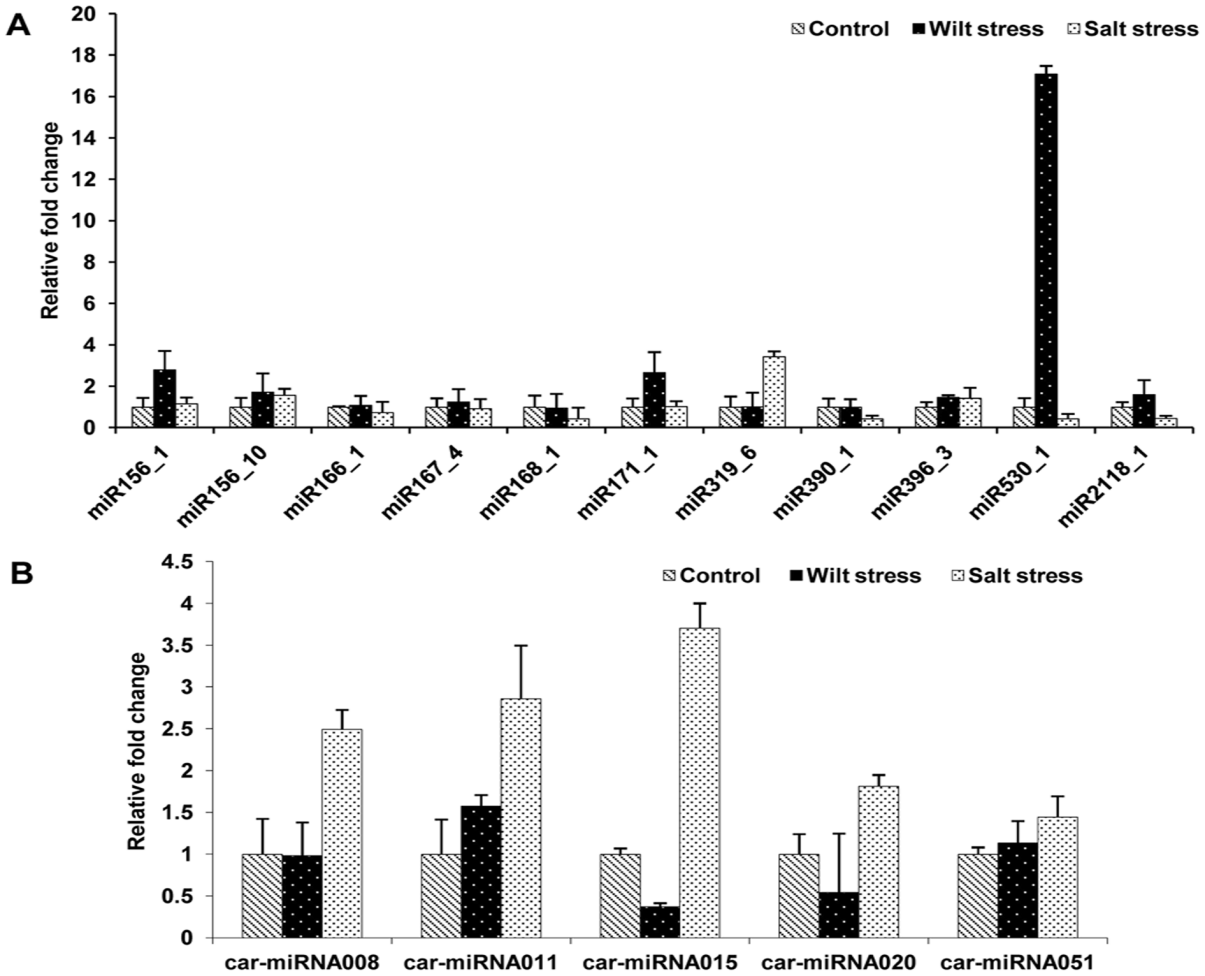
Expression analyses of selected miRNAs under wilt and salt stresses as evaluated by qRT-PCR. The relative expression levels are shown as fold changes with the standard errors (SE) of three biological replicates. (A) Expression profiling of conserved miRNAs under control, wilt and salt stress conditions. (B) Expression profiling of novel miRNAs under control, wilt and salt stress conditions.

Total RNA from the tissues of control, wilt-infected and salt-stressed plants were used to validate the miRNAs. The poly(A)RNA of these three samples was reverse transcribed into cDNA for the validation of the expression of eleven conserved and five novel miRNAs using qRT-PCR. The expression levels of the chickpea miRNAs under wilt stress were significantly altered compared with those of the control conditions. In contrast, those that were observed under salt stress did not greatly change. Among the validated conserved miRNAs, miR530 was upregulated seventeen-fold during wilt stress, suggesting that it is an important candidate miRNA that is involved in the plant wilt stress response. miR156_1 and miR156_10 were slightly upregulated under both the wilt and salt stresses. miR2118, which is one of the legume-specific miRNAs, was also upregulated by approximately 0.5-fold during wilt stress compared with the control seedlings (Figure 5a, 5b). Conversely, no significant expression patterns were detected with respect to the known miRNAs in response to salt stress in the chickpea. The expression analysis of the novel miRNAs revealed that three out of five (car-miR008, car-miR011 and car-miR015) were approximately three fold upregulated on average during salt stress (novel chickpea miRNAs have been designated as “car-miRNA” throughout the manuscript, in which “car” is an abbreviation for *Cicer arietinum*). However, the expression patterns that were observed during wilt stress revealed limited information because little significant changes occurred.

### Prediction and validation of miRNA targets in chickpea

The putative miRNA targets in chickpea were predicted using the psRNATarget program [33]. The predicted target genes (approximately 358 different transcripts) were extensively involved in different biological processes involving a large number of gene families. Some of these genes encoded TFs, DNA replication proteins and those that are involved in cellular metabolism in addition to a variety of stress response-associated proteins. The target prediction analysis revealed the involvement of some of the miRNAs in regulating metabolic processes through the target genes. In chickpea, miR159 is involved in the metabolism of amino acids, fatty acids and lipids. One of the target genes of miR159 encodes acyltransferase, which is essential for ester biosynthesis. The chickpea miR156 and miR166 target genes encode squamosa promoter-binding protein and homeobox-leucine zipper protein, respectively, as previously reported [34], [35]. Table 4 describes details of the target genes of validated miRNAs; a complete list is provided as supporting information (Table S5; Table S6). The most widely targeted class of genes is the protein kinases, which are associated with plant defense mechanisms via cell signaling-related processes. The novel car-miRNA008 targets the chalcone synthase (CHS) gene. Chalcone, which is an intermediate in flavonoid biosynthesis, is involved in natural defense mechanisms. CHS expression is also involved in salicylic acid defense pathways. car-miR2118 and car-miR5213 target two defense-response chickpea genes encoding Toll/Interleukin-1 receptor-nucleotide binding site-leucine-rich repeats (TIR-NBS-LRR). Members of the TIR-NBS-LRR gene family are genuine targets for miR2118 [24]. Additionally, miR5213 suppresses defense-response genes in Medicago. The cleavage of such transcripts as mediated by miR5213 is notably conserved in AM symbiosis-capable plants, such as *Medicago truncatula, Glycine max, Lotus japonicus, Populus trichocarpa* and *Cicer arietinum*, but not in plants for which this symbiosis is not observed, such as *Arabidopsis thaliana* [25].

**Table 4:** Predicted target genes of miRNAs in chickpea.

miRNA family	Target	Putative Functions of Predicted Targets
**Conserved miRNAs**		
mir156_1	TC12891, TC03863, TC05745,TC07318,	Squamosa promoter-binding TF family protein, SCP1-like
	TC03684, TC29077, TC07318, TC15422,	small phosphatase
	TC04572	
mir156_10	TC29077, TC15422, TC12891, TC03863,	SCP1-like small phosphatase, squamosa promoter-
	TC05745, TC03684, TC07318, TC19303,	binding protein, cationic amino acid transporter,
	TC10437, TC05493, TC18211	allantoinase 1-like protein
mir166_1	TC04758, TC15765, TC08004	ClassIII HD-ZIP, REVOLUTA
mir167_4	TC21867, TC03743, TC03697	Monosaccharide transport protein 1, MFS, tubulin-folding
		cofactor E
mir168_1	TC06138, TC16221, TC07642	GTP-binding protein, RNA binding (RRM/RBD/RNP motifs)
mir171_1	TC15816, TC01767, TC07982	HAIRY MERISTEM 3 (HAM3), cdk protein kinase, ClpX3
mir319_6	TC03909	Putative xylogalacturonanxylosyltransferase
mir390_1	TC12049, TC05305, TC19589	Protein kinase, CZF1
mir396_3	TC18749, TC21342, TC02165, TC16760,	RNA-directed DNA polymerase, NAD(P)-binding
	TC09727, TC02085	Rossmann-fold
mir530_1	TC01544, TC20787, TC01795, TC01794	Zinc knuckle protein, expressed protein
mir2118_1	TC01089, TC09480, TC00082, TC21040,	NB-ARC disease resistance protein, expressed protein,
	TC23505	TIR-NBS-LRR
**Novel chickpea miRNAs**		
car-miRNA008	TC06967, TC05545	RING/U-box superfamily protein, chalcone synthase (CHS)
car-miRNA011	TC02274, TC14659, TC17732, TC08052,	SERPIN family protein, amelogenin, RNA binding
	TC16830, TC06852, TC05883	(RRM/RBD/RNP motifs), LEA, anion channel protein family
car-miRNA015	TC17182, TC10107	Complex 1 protein (LYR family), ribosomal L23/L15e
		family protein
car-miRNA020	TC33381, TC29465, TC00653, TC28744,	TPR-like superfamily protein, ARM superfamily protein,
	TC05383	FAD/NAD(P)-binding oxidoreductase, Protein of unknown
		function (DUF1423)
car-miRNA051	TC11550, TC31151, TC21283	SMG7, HAD superfamily protein, unique electron
		transfer flavoprotein

**Figure 6:**
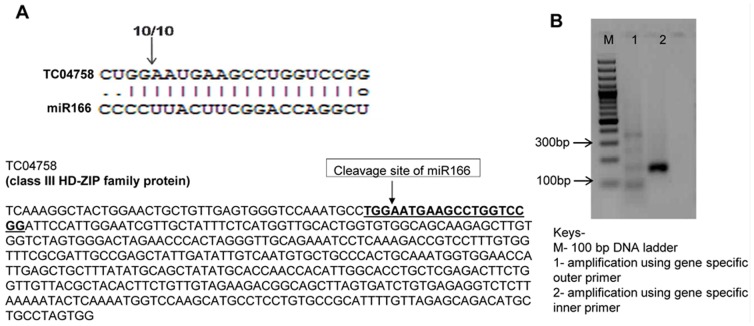
Mapping of target mRNA cleavage site of miR166 by modified 5′ RACE. The target of miR166 (TC04758) encodes a transcription factor belonging to class III of the HD-ZIP family protein. The arrow indicates the cleavage site, and the numbers above the arrow denote the frequencies of the sequenced clones.

Members of the miR166/165 family target HD-ZIP III TF genes by cleaving the mRNA at complementary base pairs in leguminous plants [34], [36], [37]. These results are similar to those of earlier predicted reports involving other plant systems. The target gene of miR166 was experimentally validated by modified 5′RLM-RACE [38], [39]. All of the positive clones were sequenced, and cleavage was observed at the 17^th^ and 18^th^ positions of the mRNA by the 5′ end of miR166 (Figure 6), unlike the previously reported miRNA-target recognition parameters [40]. Although our results are not in agreement with previous studies, such as those involving the soybean, in which miR166 target validation by 5′RACE and degradome sequencing confirmed cleavage at the 10^th^ and 11^th^ positions [41], there have been reports of the miRNA (belonging to different families)-mediated cleavage of target mRNA, thus defying the recognition rule. A total of 18 miRNA/target pairs of *Pinus taeda* possessed non-conventional cleavage sites, such as pta-miR951:AW065026, which is cleaved at the 16^th^ and 17^th^ positions [42]. Similar results have been reported in other plant species, such as mtr-miR397:AC135467 [24], ath-miR168:AGO1 [43], pvu-miR171:gi62704692 [44] and ath-miR398a:CSD1 [39]. Thus, it appears that the sequence of the target gene and the miRNA sequence determine the cleavage site apart from the conventional complimentary region-based target cleavage. Therefore, it is quite possible that chickpea has a different cleavage site for miR166 (pair miR166:TC04758) compared with other plant species.

### Analyses of GO terms and KEGG pathways

**Figure 7:**
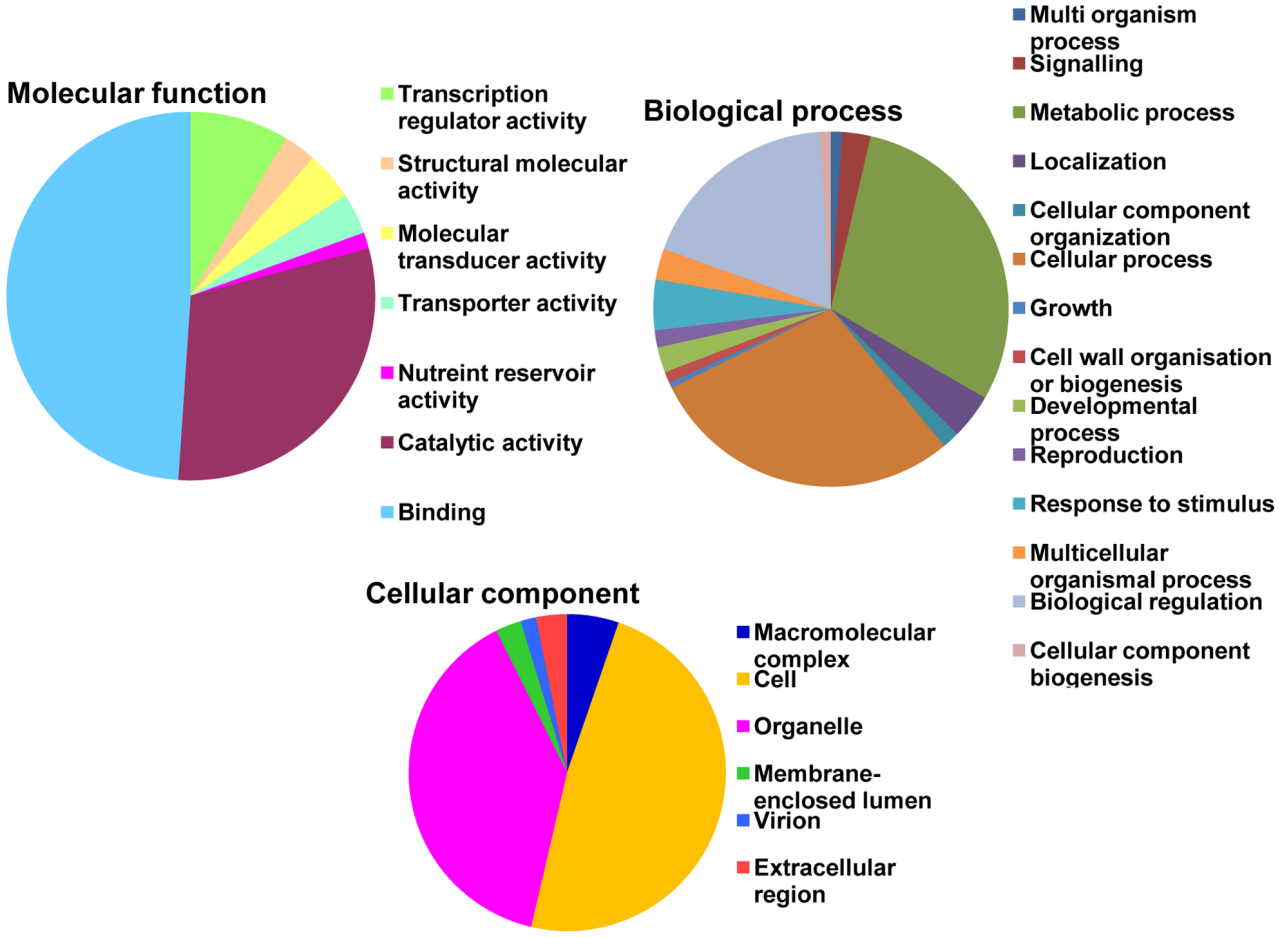
Gene ontology categories of predicted target transcripts for chickpea miRNAs. The miRNA-target genes were categorized according to the molecular function, biological process and cellular component sub-ontologies.

The GO terms of the target genes were annotated according to their biological processes, molecular functions or involvement as cellular components. The enzyme mapping of the annotated sequences was performed using direct GO for the enzyme mapping and the Kyoto Encyclopedia of Genes and Genomes (KEGG) for the definitions of the KEGG orthologs. The miRNA-targeted genes belonged to various biological processes, cellular components and molecular functions as depicted in Figure 7. The maximum numbers of target genes were involved in biological processes, including both metabolic and cellular processes. However, the target genes that were involved in binding were the most abundant (80%) within the molecular functions category.

## Discussion

In this study, high-throughput deep sequencing was used to gain in-depth knowledge of gene regulation by miRNAs in the chickpea under biotic and abiotic stresses. Salt stress is one of the major constraints to increasing chickpea productivity. Soil salinity levels affect germination in plants. Under salt stress conditions, chickpea plants show high levels of anthocyanin pigmentation in their foliage and reduced growth rates [45]. Among the biotic stresses, Fusarium wilt is one of the major soil/seed-borne diseases severely affecting chickpea growth. Its causative agent is *Fusarium oxysporum* f.sp. *ciceris*, which is a fungal pathogen.

Most of the miRNAs that were obtained in our library have a preference for the 5′-U as has been reported in other plants, which is in accordance with the defined structures of the mature miRNAs [1], [46]. The lengths of the chickpea precursors ranged from 61 to 220 nt, which were similar to those of the soybean (55 to 239 nt) and peanut (75 to 343 nt) [30], [35]. The calculation of the minimum free energy (MFE) values further added credence to these predicted hairpin structures as putative miRNA precursors. The chickpea precursors had minimum free energy values ranging from −97.2 to −26.03 Kcal/mol with an average of −50.1419 Kcal/mol, which was similar to the −50.01 Kcal/mol that was observed in *Arachis hypogaea* and the reported value of −59.5 Kcal/mol in *Arabidopsis thaliana* (Table S2) [30]. Greater increases in miRNA expression were observed following wilt stress compared with salt stress, suggesting the significant role of small RNAs in the response to pathogen attack. The total number of miRNAs was greater in the wilt stress library than in the salt stress library. Four legume-specific miRNAs were identified in the chickpea libraries, including miR2111, miR2118, miR5232 and miR5213, which were previously reported in Medicago. The sequence conservation among the different legumes and the precursor sequence similarity of these four chickpea miRNAs further substantiate their accurate identification in this study. car-miR5232 cleaves only two transcripts encoding an ATPase E1-E2 type and an expressed protein of unknown function, in concordance with a similar study in Medicago, in which miR5232 targets were experimentally confirmed by degradome sequencing [25].

The significance of miRNA* in authenticating the presence of miRNA has previously been established. A comparison of chickpea miRNA* and mature miRNA data revealed that they vary in abundance in response to the different stress treatments, which has also been previously reported [47], [48]. Our target search analysis indicated that miRNA* act upon different transcripts than do their parental miRNAs (data not shown), which has been observed in plants, animals and humans [25], [49], [50]. For example, miR393 and its miRNA* counterpart regulated the expression of genes belonging to entirely different protein families; i.e., TIR1 and SNARE, respectively [51], [52].

### Expression patterns during biotic stress

This study is the first attempt to identify miRNAs that are associated with fungal attack in the chickpea. Alterations in the expression of genes that are involved in defense during pathogen attack have been previously reported. These genes are regulated by small RNAs. miR393 was the first miRNA whose role in pathogen attack was demonstrated [51]. Eleven conserved and five novel miRNAs were analyzed in the chickpea under wilt and salt stress. Interestingly, miR530 was significantly upregulated during wilt stress. This observation suggests that its target genes are expressed at lower levels, which included the zinc knuckle proteins and microtubule-associated proteins. Zinc knuckle proteins are involved in the regulation of morning-specific growth in Arabidopsis [53]. The target of miR530 varies in different plants under different conditions and tissues. In *Populus trichocarpa*, this miRNA targets zinc knuckle (CCHC type) family proteins along with a homeobox TF [54], whereas in soybean, it targets genes that encode the CONSTANS interacting protein and nuclear transcription factor Y [55]. In *Eugenia uniflora*, miR530 targets wall-associated receptor kinase-like 14, S-acyltransferase tip-1 and a protein of unknown function in rice [56], [57]. In a recent study in maize plants that were resistant to the fungus *Exserohilum turcicum*, miR530 was identified as a novel miRNA and was predicted to target genes that are involved in kinase activities in addition to DNA-binding TFs [58]. Based on the significant upregulation of miR530 in response to Fusarium infection and its unique target genes in the chickpea, it appears to be involved in the response to pathogen attack.

The three legume-specific miRNAs (miR2111, miR2118 and miR5213) play critical roles during pathogen attack. In the chickpea, miR2111 targets a Kelch repeat-containing F-box protein. F-box proteins are responsible for the controlled ubiquitin-dependent degradation of cellular regulatory proteins and are involved in defense responses, auxin responses and floral organ development [59], [60], [61]. Targets of F-box proteins are central regulators of key cellular events and include G1 cyclins and inhibitors of cyclin-dependent kinases [62]. It appears that miR2111 and F-box proteins act together to regulate the defense response in chickpea following biotic stress. Other than F-box proteins, miR2111 also targets TIR domain-containing NBS-LRR disease resistance proteins. miR2118 and miR5213 also target the same class of R genes. TIR, which is an F-box protein, is a receptor for the plant hormone auxin [63], [64], [65], [51], and LRR consists of tandem Kelch repeats [66]. Interestingly, the chickpea miR2118 was upregulated in response to wilt infection and down regulated following salt stress. miR2118 has also been shown to be suppressed after Verticillium fungal attack in cotton [67]. Fusarium wilt leads to symptoms that are similar to those of Verticillium wilt, whose common host plant is cotton. miR2118 functions through three novel target transcripts encoding TIR-NBS-LRR disease resistance proteins, but its functional regulation remains unclear. In the soybean, miR2118 targets the protein family that is associated with disease resistance in addition to zinc finger proteins [55] and replication termination factor 2 in response to biotic (Asian soybean rust) and abiotic (water deficiency) stresses [35].

Other miRNAs also target disease resistance genes. For example, novel car-miRNA023 target proteins are involved in disease resistance. The highly conserved miRNA171 family targets more than 20 genes that are involved in different processes and pathways in the chickpea. One particular member, miR171_7, targets a disease resistance-responsive dirigent-like protein (DIR). The conspicuous involvement of disease resistance genes in the response to pathogen attack has been previously established. ESTs encoding dirigent proteins were identified in the SSH library of a chickpea that was infected with Fusarium wilt [68]. Dirigent proteins impart disease resistance through their involvement in lignification during biotic stress. Similar studies have been reported involving *Gossypium barbadense* that was infected with Verticillium fungus, in which two DIR genes were isolated from the SSH library [69].

Many of the genes that are targeted by miRNAs are involved in disease resistance and growth-related processes. Therefore, it can be surmised that these miRNAs are involved in the regulation of plant development and pathogen growth by acting both as positive and negative regulators, depending on their target genes.

### Expression patterns during abiotic stress

Our library allowed for the identification of a large number of conserved salt-responsive miRNAs, including miR390, miR172, miR171, miR169, miR408, miR159, miR396, miR2111, miR5213, miR397, miR393, miR162, miR168, miR166, miR167, miR156, miR530, miR399, miR160, miR319, miR164, miR398, miR2118 and miR394. Among these miRNAs, miR156, miR396 and miR319 were upregulated in response to salt stress, which was confirmed using qRT-PCR. Our results agreed with a previous study involving Arabidopsis, in which 10 salt-responsive miRNAs (miR156, miR165, miR319, miR393, miR396, miR167, miR168, miR171, miR152 and miR394) were reported to be involved in the high salinity stress response [70]. In the chickpea, the transcript levels of miR156 family members were elevated in response to salt stress compared with those of miR166 and others as has been reported in previous studies. Some of the miRNAs that are regulated under salt stress in other plant systems were not found in our library. This phenomenon may be due to different stages or stress conditions; i.e., particular treatment methods or species-specific responses.

Previous studies have demonstrated that miR169 family members are associated with high salt stress [71]. From our target prediction analysis, miR169-targeted genes belong to the nuclear TF family, which contains a CCAAT-binding complex. This CCAAT-binding complex is a eukaryotic promoter element that is evolutionary conserved [72]. Recent studies have demonstrated that these proteins play significant roles in abiotic stress-response pathways [39], [73]. The genes that are targeted by miR169 function in transcriptional regulation, suggesting their significant involvement in the salt stress response.

In this study, the salt-responsive miRNA miR390 explicitly targeted protein kinases and the CZF1 TF. The CZF TF is associated with intracellular signal transduction, is involved in the negative regulation of programmed cell death and responds to fungal attack via plant defense mechanisms. CZF1 contains a zinc finger with a CCCH-type domain and has been reported in *Arabidopsis thaliana* to be salt-inducible. A parallel study in upland cotton reported that the LZF TF acted in response to salt stress [74], and its network of protein-protein interactions was deduced. The chickpea miR396 exhibited higher expression levels under salt stress and was also reported to be salt-responsive in rice. Additionally, transgenic lines over expressing osa-mir396c showed reduced tolerances to salt and alkali stresses compared with wild type plants [75].

In our analysis of the miRNA expression data under both biotic and abiotic stresses, few were upregulated under both types of stresses. miR396 and a member of the miR156 family were upregulated in response to both the wilt and salt stresses at levels of approximately 1.5-fold, indicating the relative similarity between fungal infection- and salinity stress-responses in the chickpea, which was stated in a previous report, in which the chickpea responded to fungal infection (*Ascochyta blight*) more similarly to high salinity stress than to drought or cold stresses [76]. Additionally, cross talk exists between the stress-signaling pathways that involve several kinases and TFs that are important targeting candidates for several miRNAs under wilt and salt stresses [77], [78]. Our data indicate that miR172, miR319, miR171, miR390 and miR396 have serine/threonine protein kinases and MAPK protein kinases as their target genes, which involve signaling pathways. It can be presumed that together, these miRNAs might mediate defense mechanisms under stress conditions via transcriptional regulators. miRNAs also target genes that are directly or indirectly involved in the defense against various stresses. For example, car-miR08 targets a chalcone synthase gene, which is an intermediate in flavonoid biosynthesis. Flavonoids are secondary metabolites that serve variable functions, including those involving pigmentation, UV protection and antifungal defense. Therefore, it can be conferred that these miRNAs come into play during stress management in plants by targeting the genes that are involved either directly or indirectly.

The explicit role of miRNAs in regulating defense mechanisms by the complementary binding of target genes is evident through exhaustive literature reviews. This study will aid in the elucidation of the stress response mechanisms that are utilized by the chickpea. Further, there is limited available knowledge describing comprehensive studies of miRNA expression in the chickpea in response to particular stresses.

## Materials and Methods

### Plant materials and stress treatments

The chickpea cultivar ICC4958 was used throughout the study. ICC4958 is a Fusarium wilt-resistant and salt-sensitive chickpea cultivar [79], [45]. The plants of the ICC4958 cultivar were grown on a 16-h day/8-h night photoperiod cycle at 25±2°C. Fourteen-day-old seedlings were subjected to the wilt and salt stresses separately. The stress treatments were performed as follows: for wilt stress, two-week-old plants that were grown under hydroponic conditions were exposed to a toxin that was isolated from the fungus *Fusarium oxysporum* f.sp. *ciceris* for one day. For salt stress, the roots of two-week-old seedlings were immersed in a 150 mM NaCl solution for 12 h. All of the tissues (control, wilt-stressed and salt-stressed) were harvested at their respective time points, snap-frozen in liquid nitrogen and maintained at −80°C for further analyses.

### Small RNA library preparation and sequencing

Total RNA was isolated using the TRIzol reagent (Invitrogen, Carlsbad, CA, USA) according to the manufacturer's protocol. For the construction of the small RNA library, low molecular weight (LMW) RNA was enriched by the LiCl method. Equal amounts of RNA were pooled from the root and shoot tissues for each group to generate a LMW RNA library. The RNA was run on a 15% polyacrylamide gel, and the 20 to 30 nt small RNA fraction was extracted and eluted. A preadenylated adaptor was ligated to the 5′ end of the small RNAs with T4 ligase. The ligation product was eluted, and subsequently, 3′ end adaptor ligation was performed [80] followed by RT-PCR. The PCR products were checked for quality and quantified using a Bioanalyzer (Agilent, Germany). The samples were then sequenced using the Illumina Genome Analyzer IIx (Illumina Inc., USA).

### Computational sequence analysis for identification of miRNAs

The total reads were trimmed and filtered using the UEA small RNA workbench 2.4- Plant version sequence file pre-processing tool (http://srna-tools.cmp.uea.ac.uk/) [81]. The unique tags were generated following a series of processing steps, which included adaptor trimming (using the adaptor removal tool), the elimination of low-quality sequences and the removal of contaminated and other non-coding RNAs, including tRNAs, rRNAs, etc. The UEA sRNA toolkit-Plant version filter pipeline (http://srna-tools.cmp.uea.ac.uk/) was used to exclude the low-complexity and low-quality sequences and eliminate the t/r RNA population by mapping them to plant t/r RNAs from the "Rfam" database, Arabidopsis tRNAs from “The Genomic tRNA Database” and plant t/rRNA sequences from the “EMBL” release 95. Then, the miRCat pipeline (miRNA categorization) was used to predict novel miRNAs and their precursors using default parameters [82]. The secondary structures of the small RNA sequences were folded using RNAfold (http://rna.tbi.univie.ac.at/cgi-bin/RNAfold.cgi) to predict potential miRNA precursors. The small RNA sequences that had characteristic hairpin structures, together with additional minimal folding free-energy indices (MFEI) [83], [84], were considered to be candidate miRNAs by miRCat. The small RNA sequences that matched the following criteria were considered to be valid miRNA precursors: i) no more than 3 consecutive mismatches between the miRNA and miRNA*; ii) at least 17 of the 25 nt surrounding the miRNA must be involved in base pairing; iii) the hairpin must be at least 75 nt in length; and iv) at least 50% of the bases in the hairpin should be paired. The folding structures of the precursors of the new miRNA with the miRNA* were carried out using the UEA sRNA toolkit-RNA hairpin folding and annotation tool, which uses the Vienna Package to obtain the secondary structure of a precursor sequence, highlighting the miRNA/miRNA* sequences on the hairpin structure [85]. The data discussed in this publication has been deposited in Gene Expression Omnibus [86] repository under the accession number GSE57857 (http://www.ncbi.nlm.nih.gov/geo/query/acc.cgi?acc=GSE57857).

### miRNA validation by poly(A) tail assay-based quantitative real-time PCR (qRT-PCR)

The predicted chickpea miRNAs were validated by performing poly(A)-tailed RT-PCR on sixteen miRNAs, including eleven conserved and five novel miRNAs. The total RNAs from the treated and control samples were extracted using the TRIzol reagent (Invitrogen, Carlsbad, CA, USA) according to the manufacturer's instructions. A 1-µg aliquot of this RNA was used for the poly(A) tailing using the Poly(A) Tailing Kit (Ambion, USA) according to the manufacturer's instructions and then purified using the RNeasyMinElute Cleanup Kit (QIAGENGmBH, Germany). The poly(A) RNA (2 µg) was then reverse-transcribed into cDNA that was primed by a standard poly(T) anchor adaptor using an RTQ primer. For the RT-PCR reaction, the conditions were as follows: 65°C for 10 min, 4°C for 2 min, 50°C for 60 min and 70°C for 15 min. Three biological replicates per sample were used for the analyses.

The poly(T) cDNA was diluted 10-fold and used to perform qRT-PCR using KAPA FAST SYBR Green chemistry (Kapa Biosystems, USA). For the qRT-PCR, the sequences of the specific miRNAs that were validated served as the forward primer and RTQ uni-primer, having an adaptor sequence as the reverse primer (Table S7). The 5S rRNA was used as the reference gene for all of the reactions. Three biological replicates were used per sample in addition to three technical replicates along with a no-template control and no-RT enzyme control. The data were analyzed using the 2[-Delta DeltaC(T)] method [87] and reported as the means ± standard errors (SE) of three biological replicates.

### Prediction and validation of chickpea miRNA target genes

The chickpea transcript dataset, which was downloaded from the chickpea transcriptome database (CTDB), was used to determine the potential target mRNA candidates for the miRNAs using the psRNATarget program with default parameters (http://plantgrn.noble.org/psRNATarget/). To reduce the false-positive prediction rate, the cut-off threshold was set at 0 to 3.0 points. Thus, all of the sequences with ≤3.0 points were considered to be miRNA targets. The functional annotations of the predicted target transcripts were performed using the NCBI nucleic acid and protein databases. Based on the predicted data, miRNA166 was validated using modified 5′ RACE. For this validation, the FirstChoice RLM-RACE Kit (Ambion, USA) was used with minor modifications, and the cDNA amplification was carried out using 1 µg of total RNA. A single PCR fragment was cloned into the pGEM-T Easy Vector (Promega, USA) and sequenced to identify the 5′end of the target gene.

### Analyses of GO terms and KEGG pathways

The GO terms of the target genes were annotated according to their biological processes, molecular functions or involvement as cellular components using Blast2GO [88]. The enzyme mapping of the annotated sequences was performed directly using the GO terms, and the Kyoto Encyclopedia of Genes and Genomes (KEGG) was used to define the KEGG orthologs.

## Supporting Information

Figure S1 **Elimination summary of the reads.**
(TIF)

Table S1 **Conserved miRNAs that were identified in the three libraries with their detailed information.**
(XLSX)

Table S2 **Precursor sequences of conserved miRNAs with their predicted secondary structures.**
(XLSX)

Table S3 **Detailed information regarding novel chickpea miRNAs.**
(XLSX)

Table S4 **Precursor sequences of novel miRNAs with their predicted secondary structures.**
(XLSX)

Table S5 **List of the target genes that were identified for all of the conserved miRNAs.**
(XLSX)

Table S6 **List of the target genes that were identified for all of the novel miRNAs.**
(XLSX)

Table S7 **List of the primer sequences that were used in this study.**
(XLSX)
