# Supplementary figures and images for: Identification and Characterization of Wilt and Salt Stress-Responsive MicroRNAs in Chickpea through High-Throughput Sequencing
Source: PLoS One. 2014 Oct 8;9(10):e108851. doi: 10.1371/journal.pone.0108851 (PMC4190074; doi:10.1371/journal.pone.0108851)

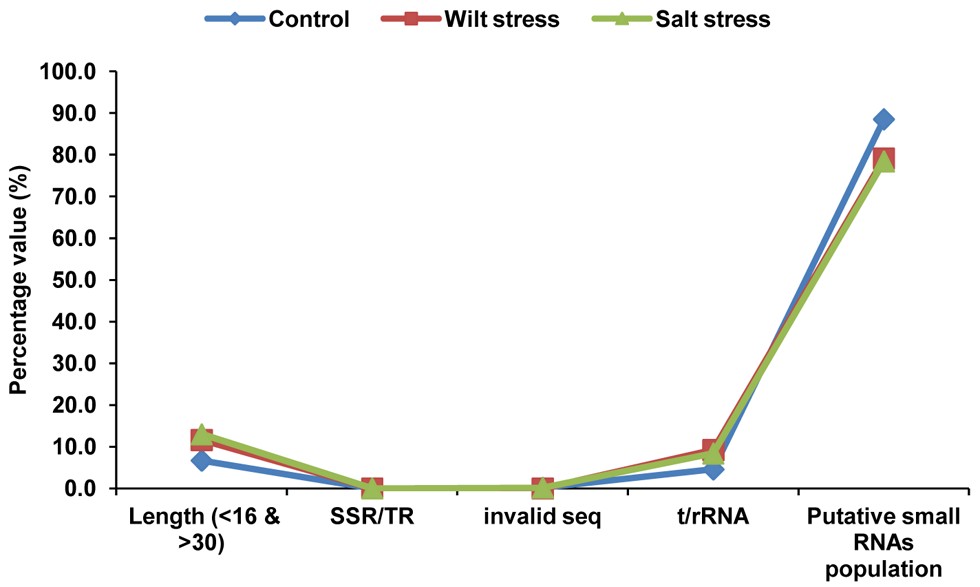

Supplement: Figure S1 — Elimination summary of the reads. (TIF) [file pone.0108851.s001.tif]
